# Long-term effects of competition and environmental drivers on the growth of the endangered coral *Mussismilia braziliensis* (Verril, 1867)

**DOI:** 10.7717/peerj.5419

**Published:** 2018-08-10

**Authors:** Felipe V. Ribeiro, João A. Sá, Giovana O. Fistarol, Paulo S. Salomon, Renato C. Pereira, Maria Luiza A.M. Souza, Leonardo M. Neves, Gilberto M. Amado-Filho, Ronaldo B. Francini-Filho, Leonardo T. Salgado, Alex C. Bastos, Guilherme H. Pereira-Filho, Fernando C. Moraes, Rodrigo L. Moura

**Affiliations:** 1 Departamento de Geologia (GGO), Universidade Federal Fluminense, Niterói, Rio de Janeiro, Brazil; 2 Instituto de Biologia, Universidade Federal do Rio de Janeiro, Rio de Janeiro, Brazil; 3 Instituto de Pesquisas Jardim Botânico do Rio de Janeiro, Rio de Janeiro, Rio de Janeiro, Brazil; 4 Departamento de Ciências do Meio Ambiente, Universidade Federal Rural do Rio de Janeiro, Três Rios, Rio de Janeiro, Brazil; 5 Departamento de Engenharia e Meio Ambiente, Universidade Federal da Paraíba, Rio Tinto, Paraíba, Brazil; 6 Departamento de Oceanografia e Ecologia, Universidade Federal do Espírito Santo, Vitória, Espirito Santo, Brazil; 7 Instituto do Mar, Universidade Federal de São Paulo, Santos, São Paulo, Brazil

**Keywords:** Cyanobacteria, Coral reefs, Sea surface temperature, Turbid zone reefs, Abrolhos, Brazil, Turbidity, *Symbiodinium*, Allelopathy, Turf algae

## Abstract

Most coral reefs have recently experienced acute changes in benthic community structure, generally involving dominance shifts from slow-growing hard corals to fast-growing benthic invertebrates and fleshy photosynthesizers. Besides overfishing, increased nutrification and sedimentation are important drivers of this process, which is well documented at landscape scales in the Caribbean and in the Indo-Pacific. However, small-scale processes that occur at the level of individual organisms remain poorly explored. In addition, the generality of coral reef decline models still needs to be verified on the vast realm of turbid-zone reefs. Here, we documented the outcome of interactions between an endangered Brazilian-endemic coral (*Mussismilia braziliensis*) and its most abundant contacting organisms (turf, cyanobacteria, corals, crustose coralline algae and foliose macroalgae). Our study was based on a long (2006–2016) series of high resolution data (fixed photoquadrats) acquired along a cross-shelf gradient that includes coastal unprotected reefs and offshore protected sites. The study region (Abrolhos Bank) comprises the largest and richest coralline complex in the South Atlantic, and a foremost example of a turbid-zone reef system with low diversity and expressive coral cover. Coral growth was significantly different between reefs. Coral-algae contacts predominated inshore, while cyanobacteria and turf contacts dominated offshore. An overall trend in positive coral growth was detected from 2009 onward in the inshore reef, whereas retraction in live coral tissue was observed offshore during this period. Turbidity (+) and cyanobacteria (−) were the best predictors of coral growth. Complimentary incubation experiments, in which treatments of *Symbiodinium* spp. from *M. braziliensis* colonies were subjected to cyanobacterial exudates, showed a negative effect of the exudate on the symbionts, demonstrating that cyanobacteria play an important role in coral tissue necrosis. Negative effects of cyanobacteria on living coral tissue may remain undetected from percent cover estimates gathered at larger spatial scales, as these ephemeral organisms tend to be rapidly replaced by longer-living macroalgae, or complex turf-like consortia. The cross-shelf trend of decreasing turbidity and macroalgae abundance suggests either a direct positive effect of turbidity on coral growth, or an indirect effect related to the higher inshore cover of foliose macroalgae, constraining cyanobacterial abundance. It is unclear whether the higher inshore macroalgal abundance (10–20% of reef cover) is a stable phase related to a long-standing high turbidity background, or a contemporary response to anthropogenic stress. Our results challenge the idea that high macroalgal cover is always associated with compromised coral health, as the baselines for turbid zone reefs may derive sharply from those of coral-dominated reefs that dwell under oligotrophic conditions.

## Introduction

Coral reef ecosystems are facing a sharp loss of biodiversity and habitat structure (Bruno et al., 2009; Hoegh-Guldberg, 1999). At the global scale, this decline is associated with thermal anomalies and ocean acidification, which affect coral fitness through bleaching and reduced calcification rates (e.g., Veron et al., 2009). Local and regional level drivers associated with anthropogenic impacts also play a major role in reef degradation (Hughes, 1994). As watersheds become degraded from poor land-use practices and urbanization, rivers deliver increased sediment loads, as well as industrial, agricultural, deforestation and domestic by-products (Doney, 2010). Increased nutrification and sedimentation may have a severe impact on coral reefs, which most often thrive in meso- and oligotrophic tropical shallow waters (Sanders & Baron-Szabo, 2005). Locally, overfishing promotes an overall trophic downgrading of the coral reef, with severe consequences to ecosystem functioning (Edwards et al., 2013).

Increased sediment load and turbidity, either from dredging or terrigenous/riverine sources, impact reef organisms by smothering and altering light regimes, inherently changing community structure and net productivity (Rogers, 1990). Coral responses to sedimentation comprise decreases in live tissue, growth rates and skeletal density. Lowered recruitment, diversity and species richness at the assemblage level also result from overgrowth by macroalgae and cyanobacteria (Fabricius, 2005). In addition, wastewater discharges often carry organic and inorganic compounds from industry and agricultural fertilizers, as well as trace metals that affect coastal reefs (Doney, 2010). Nutrient enrichment is likely followed by acute changes in benthic community composition and abundance, including mortality of less tolerant taxa, and increased cover of turf, cyanobacteria and macroalgae (Albert et al., 2005). Higher levels of dissolved organic carbon (DOC) and particulate organic matter (POM) disrupts coral microbiomes, leading to outbreaks of pathogens, sloughing and death (Kline et al., 2006).

Overfishing of large herbivorous fish contributes to algal outbreaks and increases in DOC concentrations (Edwards et al., 2013). In addition, fishing pressure on top predators may increase disease prevalence through the predation release of lower level vector species (Raymundo et al., 2009). Once the overall biomass and size of higher trophic levels have sufficiently decreased, the system undergoes cascading effects that may disable the top down control of the entire benthic community, ultimately resulting in the loss of biodiversity and ecosystem services (Mumby et al., 2006). Such cumulative effects of multiple stressors have driven several reefs toward a phase-shift from a coral dominated environment to ephemeral soft-coral, turf and macroalgae dominance (Done, 1992; Mumby et al., 2006). Since successive perturbations can disrupt ecological interactions, a degradation loop takes place, releasing algae from competition and predation, allowing less palatable forms to take over, ultimately preventing coral populations from being replenished (Done, 1992; Hughes, 1994).

Cyanobacteria are important players in phase-shifting reefs, growing as dense mats and tufts, as well as part of the so-called turf consortia (Connell, Foster & Airoldi, 2014). The abundance of cyanobacteria in reef systems is positively correlated with eutrophication and thermal anomalies (Taylor et al., 2014). When in direct contact with corals, cyanobacteria may disrupt the microbial community within the coral mucus biofilm and trigger an exacerbated growth of pathogens (Morrow et al., 2011). Cyanobacteria are known to inhibit coral recruitment (Kuffner et al., 2006) and to produce toxins and enzymes with grazing deterrence properties (Smith et al., 2010). Despite their relatively ephemeral/opportunistic nature, cyanobacteria can render permanent impacts on corals by overgrowth and progressive deterioration of live tissue (Bender, Diaz-Pulido & Dove, 2012), shading and abrasion (McCook, Jompa & Diaz-Pulido, 2001), as well as allelopathy (Rasher et al., 2011). In addition, they can affect coral larvae settlement (Box & Mumby, 2007) and attract larvae to ephemeral surfaces (Vermeij et al., 2009).

Long-term (i.e., decadal) data series are lacking for most regions, despite being needed to assess the dynamics of coral reef cover, because scleractinians grow at a few centimetres per year and climate-oceanographic forcing may operate either episodically or in cycles deviating from seasonal oscillation (Hughes, 1994). In addition, percent cover data from transects, which are usually employed in reef monitoring (e.g., Shuman, 2007), may not allow for disentangling the outcomes of coral competition with their surrounding faster-growing organisms. For instance, the negative effect of cyanobacteria on living coral tissue may not be detected from estimates of percent cover gathered with randomly distributed transects, as these ephemeral organisms tend to be replaced by longer-living canopy-forming macroalgae, or complex turf-like consortia structured by articulated calcareous and other algae (Titlyanov & Titlyanova, 2009). Here, we circumvented this bias by sampling fixed coral colonies over 11 years.

Brazilian reefs are characterized by high endemism levels within low diversity assemblages subjected to high turbidity and heavy terrigenous sedimentation (Laborel, 1969). Therefore, the Eastern South American coast comprises several notable examples of turbid-zone reefs in the tropical West Atlantic (see Moura et al., 2016), departing from the archetypical Caribbean/Indo-Pacific healthy-reef model (high coral-low algal cover) (Perry & Larcombe, 2003). Turbid zone reefs, traditionally perceived as marginal habitats for healthy coral growth, occupy large areas, grow as fast as oligotrophic reefs, and often support high coral cover (Morgan et al., 2017). In addition, corals from turbid-zone reefs may be more effective in sediment sloughing and in the concurrent use of phototropic/heterotrophic feeding, besides being more resistant and resilient to thermal anomalies (Anthony, 2006; Morgan et al., 2017).

Despite such characteristics, coastal development, overfishing and climate changes deeply affected the community structure of Brazilian reefs in the last decades (Leão & Kikuchi, 2005; Dutra, Kikuchi & Leão, 2006). For instance, fisheries yields and fish biomass already show sharp declines in the Abrolhos Bank (Francini-Filho & Moura, 2008; Freitas et al., 2011), a region that comprises the largest, richest and best-protected reefs in the South Atlantic (Laborel, 1969; Moura et al., 2013). Turf algae cover has increased across the region (Francini-Filho et al., 2013), whereas one of the main endemic reef corals, *Mussismilia braziliensis*, a stress-tolerant Neogene relic, is predicted to be nearly extinguished in less than a century if the current rate of mortality due to diseases is not reversed (Francini-Filho & Moura, 2008; Bruce et al., 2012). The Abrolhos reefs are distributed along a cross-shelf gradient of terrigenous influence and fishing effort (Moura et al., 2013), providing a propitious context to study the relative effects of coastal influence and protection. Here, we present the results of an 11-year survey of sixteen *M. braziliensis* colonies and their neighbouring organisms, complemented by short-term incubation experiments with the symbionts of *M. braziliensis*’ (*Symbiodinium* spp.) subjected to competitors’ exudates. Our study addresses the coupling between environmental drivers and competitive processes in the contact zone between corals and other organisms.

## Material and Methods

### Study Site

**Figure 1:**
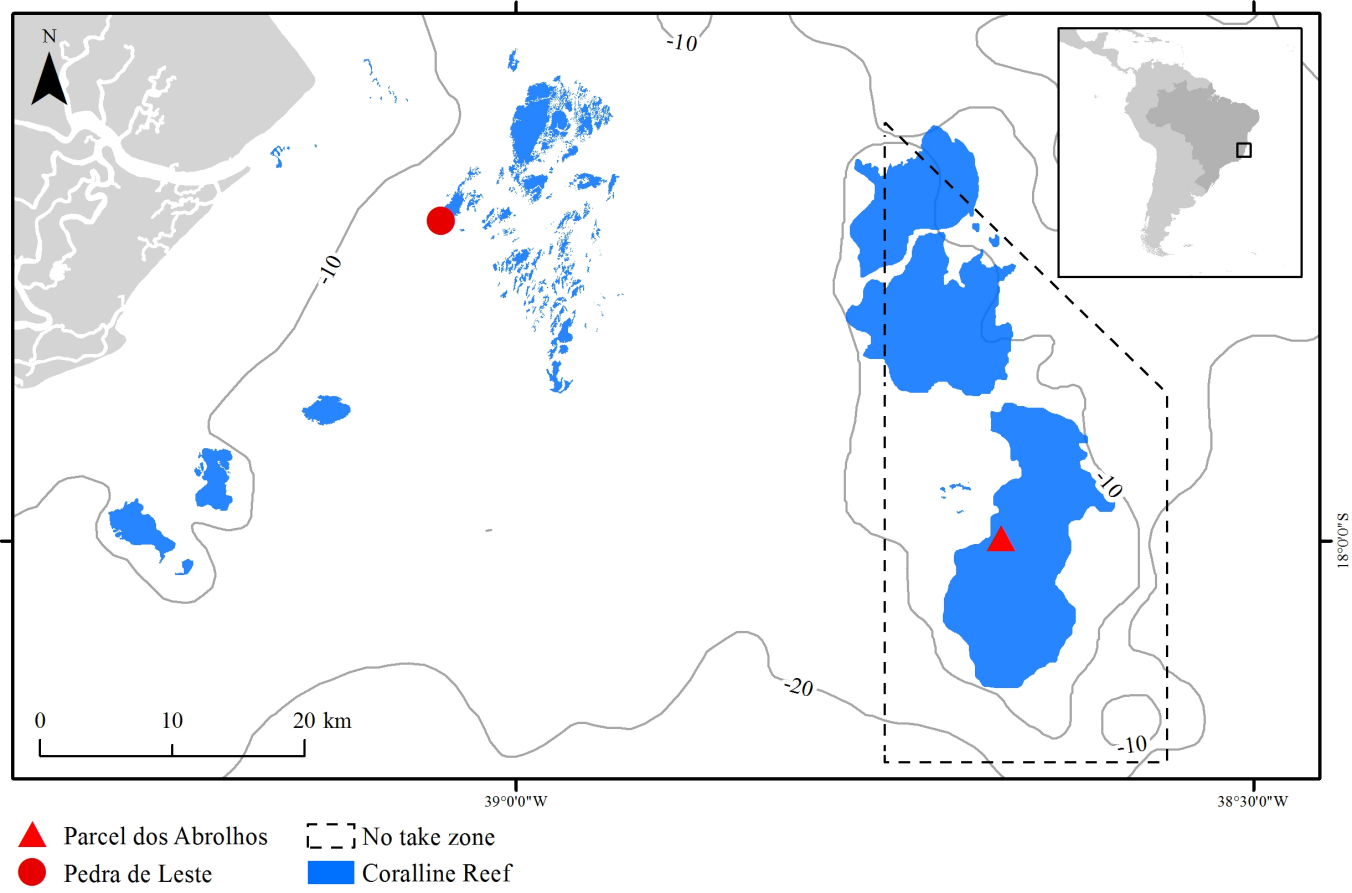
The Abrolhos reefs off Southern Bahia, Brazil. Depth contours and the Abrolhos National Marine Park no-take zone are represented as solid and dashed lines, respectively. Sampling sites and coralline reefs are shown in red and blue, respectively.

The Abrolhos Bank (16°40′–19°40′ S, 39°10′–37°20′ W) comprises a 40,000 km^2^ benthic mosaic of reefs, rhodolith beds and unconsolidated sediments, encompassing the largest and richest reefs within the South Atlantic (Moura et al., 2013). The reefs are remarkable for their mushroom-shaped pinnacles with flat tops, which result in strong habitat variation at relatively small spatial scales (pinnacles’ walls and tops) (Bastos et al., 2008). Pinnacles are dominated by massive and encrusting corals, reaching up to 30% of the benthic cover (Francini-Filho et al., 2013). Most corals are Brazilian-endemic, and there is an overall lack of branching forms, except for milleporids (Laborel, 1969; Moura et al., 2016). The studied species, *M. braziliensis*, is restricted to the shallow (5–10 m depth) pinnacles’ tops, where it is regarded as one of the most important reef builders (Leão & Kikuchi, 2005, but see Bastos et al., 2008) and covers up to 10% of the reef (Francini-Filho et al., 2013). Sampling was carried out in one inshore unprotected reef (Pedra de Leste—PLES) and in one offshore no-take reef (Parcel dos Abrolhos—PAB), the latter within the Abrolhos National Marine Park (Fig. 1). When compared to the offshore reef, the inshore reef has lower fish biomass (Francini-Filho et al., 2008) and higher turbidity, sedimentation, nutrient, DOC levels and microbial loads (Segal et al., 2008; Bruce et al., 2012), as well as a higher cover of fleshy macroalgae (mostly *Dictyota* spp.) reaching up to 20% of the reef tops compared to less than 5% on the reef tops found offshore (Francini-Filho et al., 2013).

### Sampling and sample processing

**Figure 2:**
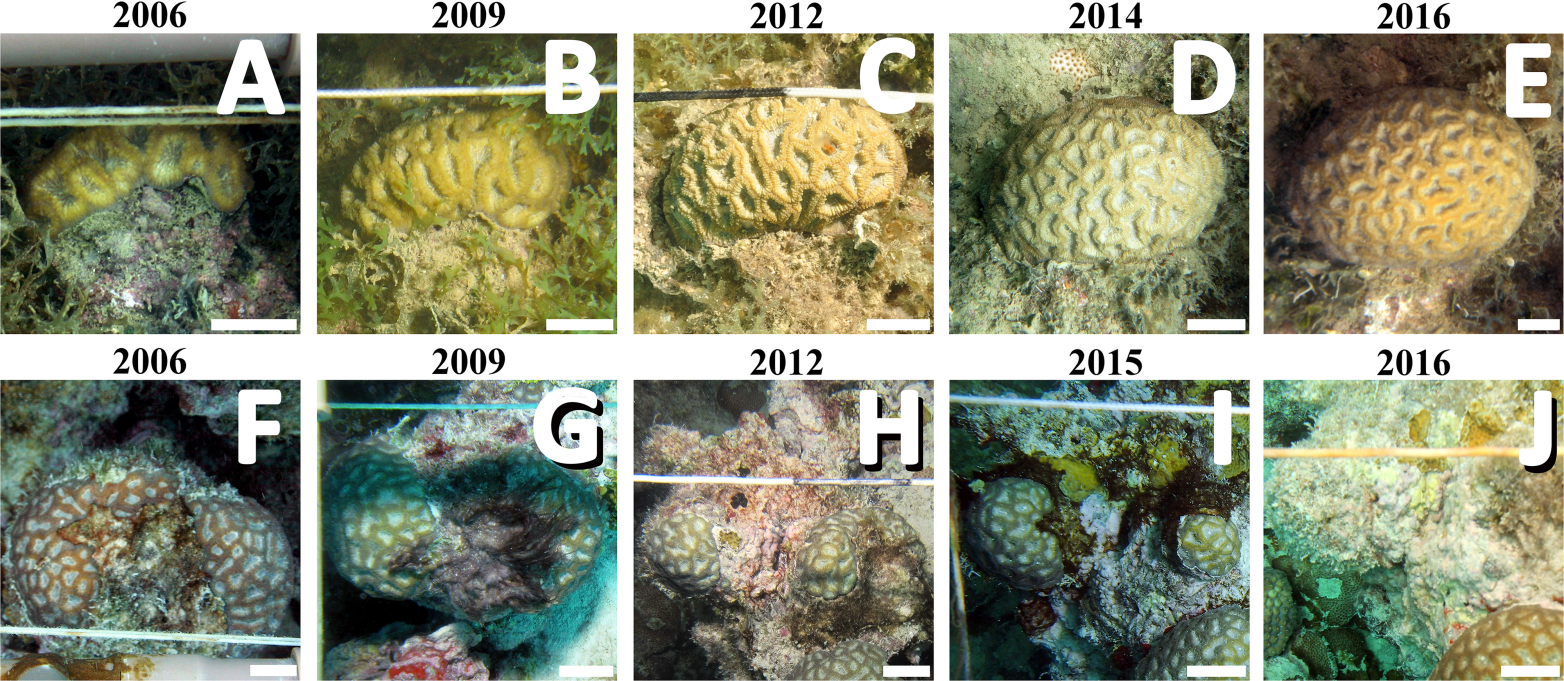
Sequential images from individual colonies in the inshore (PLES: A–E) and offshore (PAB, F–J) reefs. Images show the contrasting growth trajectories of the corals and turf/cyanobacteria dominance in their perimeters. Scale bars = 2 cm. Photographs taken by the authors.

Coral colonies (*n* = 16, 8 from PLES and 8 from PAB) were selected from the database of a long-term monitoring program with fixed photo-quadrats, based on the completeness and quality of the time series. Sampling was carried out yearly during the austral summer (January–March), from 2006–2016. No data are available for 2007, 2010 and 2011 (both sites), and from 2008 (PLES). The living area of each coral colony and the perimeter in contact with each organism were measured with ImageJ software (Schneider, Rasband & Eliceiri, 2012). Categories of surrounding organisms included the most abundant functional groups: turf algae, cyanobacteria, corals, crustose coralline algae (CCA) and foliose macroalgae. Turf algae comprise a matrix of fine-branched filamentous macroalgae of distinct taxa interwoven within 2–5 cm thick mats (Connell, Foster & Airoldi, 2014), while cyanobacteria appeared as homogeneous brown-red mats and tufts (Fig. 2). Coral growth (planar area change, which is not equivalent to liner extension) and changes in the relative perimeter of surrounding organisms were estimated from the difference between measurements from each sampling period.

### Environmental data

Summer sea surface temperatures (SSTs) (4 µnighttime, January–March) and turbidity data (Diffuse Attenuation Coefficient at 490 nm—Kd 490) for both reefs were extracted and processed with SeaDas software (ver. 7.4) using level-3 imagery from the MODIS sensor (https://oceancolor.gsfc.nasa.gov) onboard the Terra satellite, using a monthly compositing period and 9 km spatial resolution.

### Allelopathy experiments

Cyanobacterial outbreaks can be harmful to corals and were recently recorded in Abrolhos (Ribeiro et al., 2017). In order to investigate their effects over corals we ran incubation experiments with the symbionts from *M. braziliensis*’ (*Symbiodinium* spp.) subjected to cyanobacterial exudates obtained from cultures of the most common filamentous cyanobacterial morphotype found in Abrolhos, cf. *Lyngbya majuscula*. Symbionts (Culture Collection of Microalgae at UFRJ - CCMR 0100) and cyanobacteria (CCMR 0174, 0175, 0176) were collected in the Abrolhos reefs and cultivated at the Laboratory of Marine Phytoplankton, Federal University of Rio de Janeiro. Both, the symbionts and the cyanobacteria were maintained at 26 °C, under a 16:8 h light:dark period (ca. 50 µmol photons m^−2^ s^−1^). Three cyanobacterial isolates (*n* = 3, plus control) were tested to account for possible intraspecific variation in metabolite concentration. The cyanobacterial cultures were left growing for one month, and then 50 mL aliquots were extracted and filtered with Whatman^®^ glass microfiber filters (GF/F, 25 mm). The filtrate from each cyanobacterial culture was than distributed into quadruplicate 20 mL glass vials containing 9 mL of symbiont culture in f/2 medium. Each vial of treatments and control incubations received 9 ml of cyanobacterial exudate. Controls were made by adding f/2 medium to the symbiont cultures. Two millilitres of f/2 medium were added to the treatments in order to avoid nutrient limitation. After addition of cyanobacterial exudates and f/2 medium, each experimental unit had an initial symbiont concentrations of 6 ×10^4^ cells mL^−1^. The experiment was run for 72 h with the same temperature and irradiance as described above. Cell counts were performed daily with a BD Accuri™ C6 flow cytometer using the chlorophyll fluorescence and forward-scattered light as discrimination parameters to detect *Symbiodinium* cells. Daily values were normalized using time zero concentrations.

### Statistical analyses

A distance-based linear model (DISTLM; Legendre & Anderson, 1999; McArdle & Anderson, 2001) was used to investigate the relationships between coral growth and its possible predictors (turf algae, cyanobacteria, foliose macroalgae, CCA, coral, temperature and turbidity), using different time lags for temperature and turbidity (0, 1 and 2 years). We retained only the 1-year lag in subsequent models (i.e., using temperature and turbidity from the previous year), as it presented the highest explanatory power. Distance offshore was not incorporated in the models due to its colinearity with turbidity. The most significant predictors were selected using the best selection procedure, and the Akaike’s information criterion (AIC) was used to select the most parsimonious models. A distance-based redundancy analysis (dbRDA, Legendre & Anderson, 1999; McArdle & Anderson, 2001) was used to quantify the associations between predictors and colony area change. Multiple partial correlations of the selected predictors according to DistLM analysis with the dbRDA axis were also examined in order to interpret the relationship and identify the dominant forces driving the coral growth response to the environmental variables. PERMANOVA pairwise tests were performed in order to discriminate differences in coral growth among years, as well as the frequency of contact with neighbouring organisms between sites. Analyses were run with software Primer 6 (Anderson, Gorley & Clarke, 2008).

Permits for the field study were granted by the Abrolhos National Park (SISBIO permits number 49667-1, 50872-2, 51670-2).

## Results

**Figure 3:**
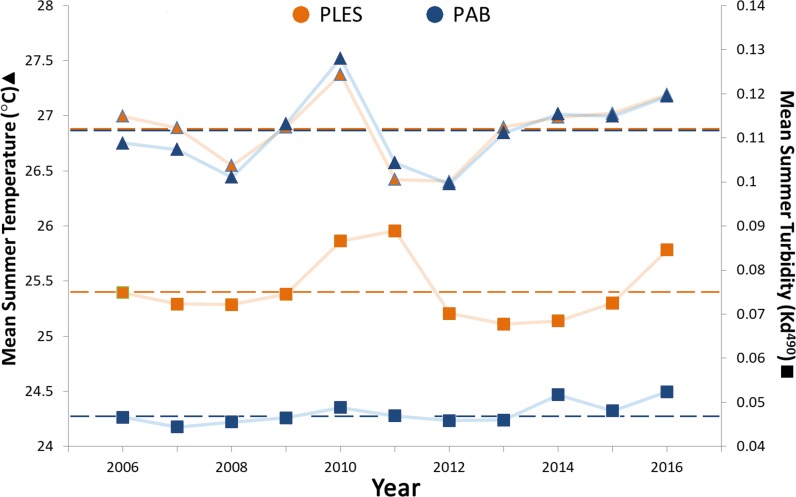
Summer sea surface temperatures and turbidity for the inshore (PLES) and offshore (PAB) reefs during the study period (2006–2016). Triangles represent temperatures and squares represent turbidity. Dashed horizontal lines represent average values.

Mean summer SSTs ranged from 26.4 to 27.5 °C during the 11 year survey period and presented only slight spatial variation, with less than 0.2 °C differences between the two sites, whereas turbidity (Kd 490) in PLES (inshore) was 1.5 times higher on average than that recorded in PAB (offshore). Turbidity spikes in PLES occurred in 2010, 2011 and 2016. Positive temperature anomalies with mean summer SSTs up to 0.5 °C above the study period average were recorded in 2010 (Fig. 3).

**Figure 4:**
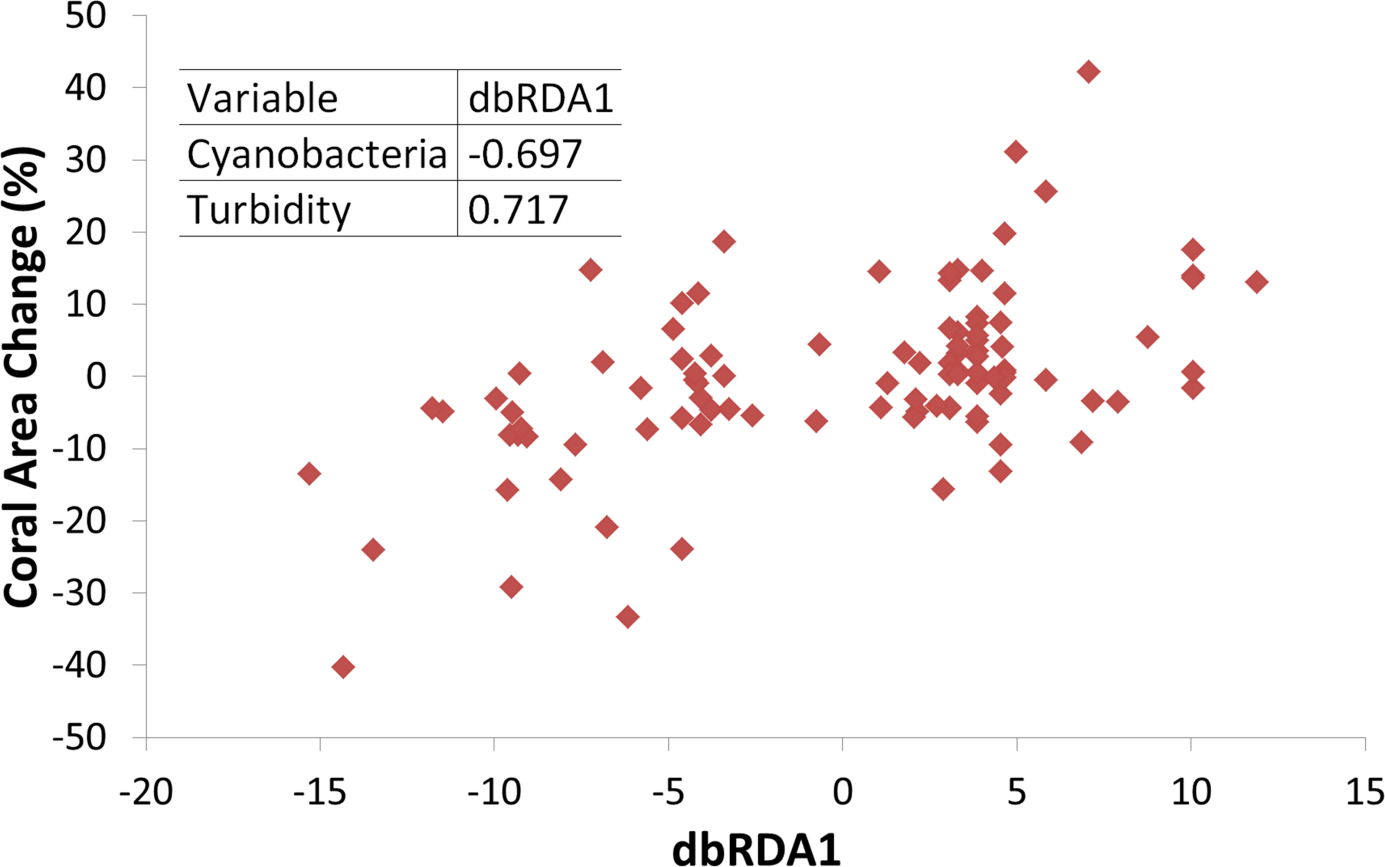
Coral growth response relationship with the first Distance-based Redundancy Analysis (dbRDA) axis, related to turbidity and cyanobacteria perimeter. Insert shows Pearson correlation coefficients.

Turbidity and the coral perimeter in contact with cyanobacteria were significant predictors of coral growth, accounting for 27.5% of coral growth variation (Table 1). Turbidity was positively correlated with the first dbRDA axis, indicating a positive association with coral growth, while cyanobacteria was negatively correlated (Fig. 4).

**Table 1:** DistLM marginal test results and model selection.

**DistLM marginal test**					
Variable	Variable	SS(trace)	Pseudo- *F*	*P*	Prop.
1	Turf	24.055	0.16538	0.678	0.0016
2	Cyano	1,929.7	15.29	0.001	0.134
3	Dicty	61.884	0.42658	0.495	0.0042
4	CCA	240.7	1.6801	0.205	0.0167
5	Coral	123.31	0.85364	0.382	0.0085
6	Turbidity	2,068.7	16.576	0.001	0.143
7	Temperature	49.772	0.3428	0.568	0.0034
**Overall best solution**					
AIC	*R*^2^	RSS	No. Of Variables	Selections	
474.62	0.27512	10,456	2	2;6	

**Figure 5:**
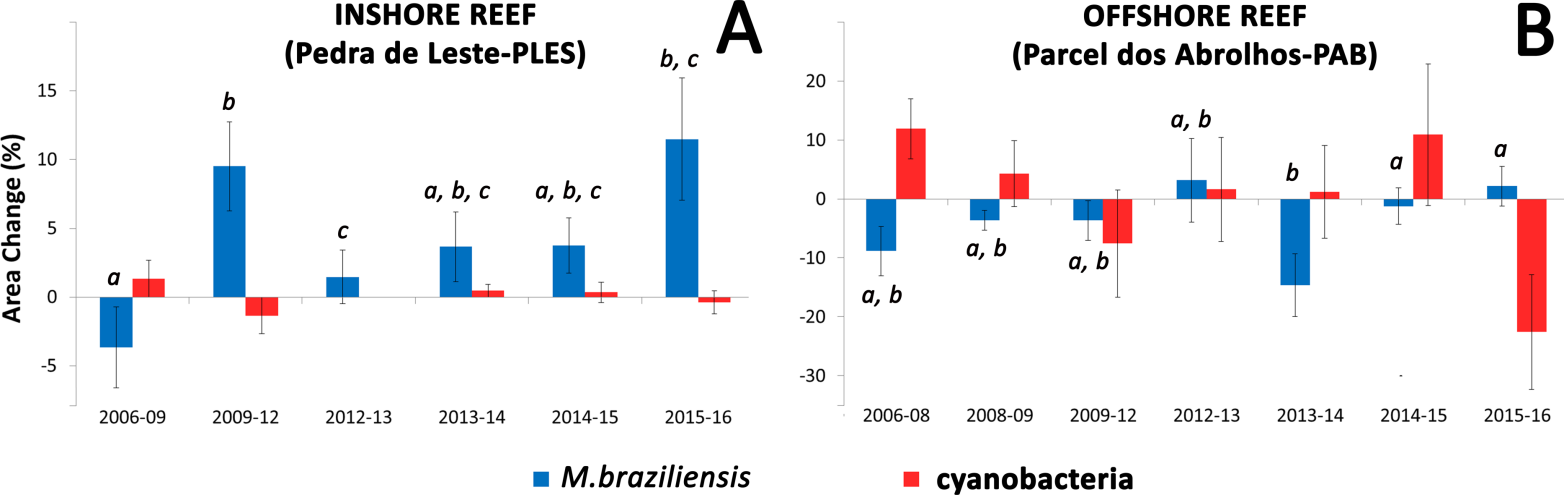
Mean values of coral and cyanobacteria area change at the inshore (A) and offshore reef (B). Letters show significant differences (PERMANOVA) for pairwise comparisons of *M. braziliensis* area change between each sampling time.

Coral growth responded negatively to direct contact with cyanobacteria (Figs. 4 and 5) and contact length with surrounding organisms was significantly different between reefs (Table 2). Over the entire study, coral-algae contacts predominated inshore, while cyanobacteria and turf dominated the contacts with corals in the offshore reef. An overall trend of positive coral growth was detected from 2009 onwards in the inshore reef, whereas retraction in live coral tissue was observed offshore during this same period. The PERMANOVA pairwise comparisons confirmed statistical differences in coral growth between years and sites (*p* < 0.05) (Table S1), as well as in the frequency of cyanobacteria, foliose macroalgae and CCA contacting coral colonies (Table 2).

**Table 2:** Mean extension of contacts with *M. braziliensis* colonies (2006–2016) and PERMANOVA pairwise tests results contrasting inshore and offshore samples.

Variable	PLES (%, ±SD)	PAB (%, ±SD)	*t*	*P*	Permutations
Turf	12.62 (±9.92)	14.55 (±8.40)	0.7887	0.4349	9,582
Cyanobacteria	0.10 (±0.42)	3.90 (±5.37)	4.8866	0.0001	9,364
Foliose macroalgae	6.89 (±6.35)	0.02 (±0.14)	8.4528	0.0001	9,481
CCA	0.83 (±2.10)	4.68 (±5.50)	4.3167	0.0002	9,372
Coral	2.16 (±3.51)	1.23 (±1.77)	1.8772	0.0661	9,021

**Figure 6:**
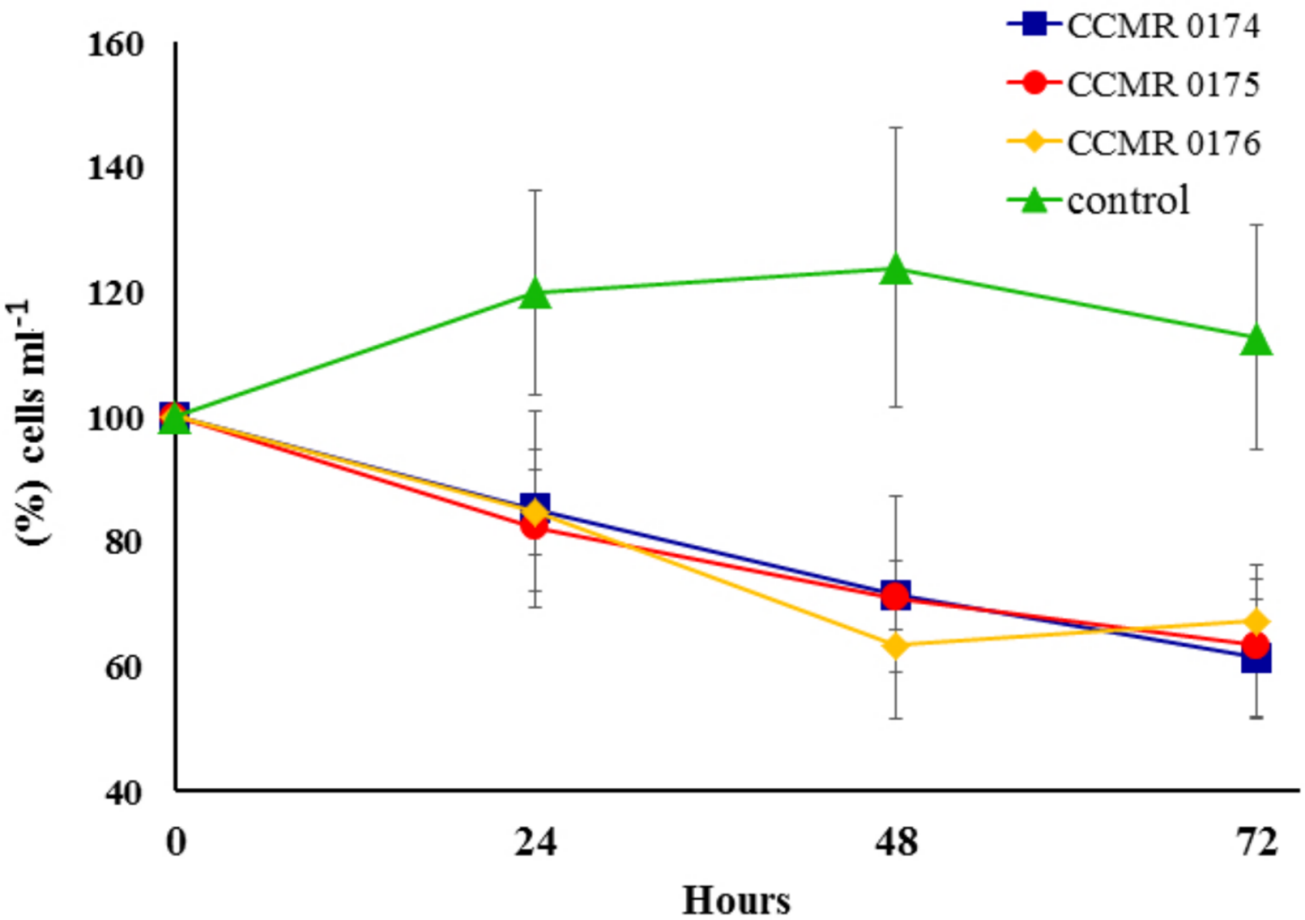
Effect of three cyanobacterial exudates on *Symbiodinium* spp. cultivated from Abrolhos’ specimens of *M. braziliensis*. Bars represent SE. Codes refer to the catalogue numbers of the Culture Collection of Microalgae at the Universidade Federal do Rio de Janeiro (CCMR-UFRJ).

Exudates harvested from the media of cyanobacteria cultures affected symbiont growth during incubations. Percent variation of *Symbiodinium* cell concentrations in treated vials dropped consistently during the 72 h incubations, whereas in untreated vials it increased for the first 48 h, and slightly dropped after 72 h, but always had higher concentrations compared to the exudate-treated vials (Fig. 6). Cell counts in vials with cyanobacteria exudates had a negative variation of 1.0.10^4^ cells ml^−1^ after 24 h, 2.0.10^4^ cells ml^−1^ after 48 h and 2.3.10^4^ cells ml^−1^ after 72 h, whereas controls showed a positive variation of 1.4.10^4^, 1.6.10^4^ and 1.0.10^4^ cells ml^−1^, respectively.

## Discussion

Turbid zone reefs, often considered marginal sites for coral communities, may harbour a significant cover of fast-growing and/or stress-tolerant coral species within low diversity assemblages (Anthony, 2006; Cacciapaglia & Van Woesik, 2016; Morgan et al., 2017). Rather than being local or “marginal” features, these reefs cover vast and yet unmapped areas of invaluable coralline habitat in several ocean basins (Kleypas, 1996; Moura et al., 2016; Bastos et al., 2008). Understanding the dynamics of turbid-zone reefs is essential to evaluate the generality of the models explaining the decline of coral reefs, which often involve a positive feedback loop of DOC, disease, algae, and microorganisms (DDAM model, Haas et al., 2016), among other climate and anthropogenic stressors (Hughes, 1994). The Abrolhos Bank is a foremost example of turbid zone reef (Leão & Ginsburg, 1997), with a strong cross-shelf gradient of turbidity and sedimentation, related to coastal/riverine inputs and seasonal resuspension of autochthonous fine sediments during winter intrusions of polar fronts (Leão & Kikuchi, 2005; Segal et al., 2008).

Our DistLim model evidenced that cyanobacteria and turbidity were the best predictors of coral growth in Abrolhos (see Table 1), the former presenting a negative effect. Conversely, turbidity (Kd490) was associated with positive changes in living coral tissue. For instance, the larger positive change in net coral growth in the inshore reef (2009–12) followed the 2010–11 turbidity peak (Fig. 5), suggesting improved conditions for the corals. Despite the higher nearshore turbidity and sedimentation, there was no significant cross-shelf difference in SST (Fig. 3). Indeed, we failed to detect an effect of SST on *M. braziliensis* growth, evidencing a stronger influence of the turbid regime and competition with cyanobacteria. Turbidity inherently alters light penetration and reduces the incidence of damaging UV radiation. Akin to shading (McCook, Jompa & Diaz-Pulido, 2001), this widespread “sunblock effect” in turbid zone reefs may interact antagonistically with other disturbances to alleviate the impact of coral bleaching during severe thermal stress (Guest et al., 2016; Morgan et al., 2017). In addition, higher nutrient availability may contribute to enhanced growth of *M. braziliensis*, possibly related to heterotrophic feeding, and previous studies have demonstrated increased reproductive output on inshore reefs of the Abrolhos Bank (Pires, Segal & Caparelli, 2011). However, increased sedimentation and nutrification from land-based sources may have a detrimental effect on coral growth. Indeed, corals may increase growth under physiological stress and display a “maximum-accretion-to-turnoff” response (Wooldridge, 2014). Under such circumstances, skeletal density may decrease and extension rates may increase until abrupt cessation of growth.

Benthic cyanobacteria produce a broad array of secondary metabolites, with several bioactive compounds that interfere with bacterial *quorum sensing* and coral diseases (Engene et al., 2011; Puglisi et al., 2014). Such compounds could also be cytotoxic and exert allelopathic effects in corals (Morrow et al., 2011). Contact with chemically defended algal species may trigger immune responses in *Symbiodinium*, depending on exposure, length and specific activities of secondary metabolites (Shearer, Snell & Hay, 2014). Here we present evidence of an inhibitory effect of compounds exuded by filamentous cyanobacteria over *ex hospite Symbiodinium* isolated from *M. braziliensis*. Cyanobacteria affect dinoflagelates’ development and reduce their *in hospite* density and photochemical efficiency, ultimately impairing coral growth (Titlyanov, Yakovleva & Titlyanova, 2007). Therefore, the negative effect of cyanobacteria exudates over the symbionts may play a major role in coral tissue necrosis, adding to the deleterious effects of small-scale oxygen depletion in contact zones (Haas et al., 2016). Although cyanobacteria density is positively correlated with nutrient availability from coastal sources (Ahern et al., 2007), both inshore and offshore Abrolhos’ sites have similar DOC and total nitrogen concentration (Bruce et al., 2012). Therefore, the recent cyanobacterial outbreaks, along with microbialization and diseases reported across the region (Ribeiro et al., 2017; Bruce et al., 2012; Francini-Filho & Moura, 2008), may have not been driven solely by nutrification. The allelopathic effect observed is consistent with the negative effect of cyanobacteria on coral growth shown by the DistLim model (Table 1). While water temperature had a limited effect over the long-term *M. braziliensis* growth trends, it may trigger cyanobacterial outbreaks and indirectly affect coral health (Ribeiro et al., 2017)

The cross-shelf trend of decreasing turbidity and macroalgae abundance suggests either a direct positive effect of turbidity on coral growth, or from the higher inshore cover of foliose macroalgae that constrain cyanobacterial abundance, such as recorded in the offshore reef site. Altered light regimes may lead to shifts in cyanobacterial abundance (Castenholz & Garcia-Pichel, 2013). When compared to primary succession organisms (e.g., turf and cyanobacteria), foliose macroalgae establish relatively stable assemblages with dense canopies (Steneck & Dethier, 1994), which may be considerably resistant to herbivory (Francini-Filho et al., 2010). Although macroalgae are able to compete and damage coral tissue (Tanner, 1995; McCook, Jompa & Diaz-Pulido, 2001), their negative effects over corals may be less important than those from chemically-driven cyanobacterial outbreaks. In addition, macroalgae may provide shelter for coral recruits (McCook, Jompa & Diaz-Pulido, 2001) and control epiphytism of cyanobacteria, ultimately rendering reduced contact of toxic filaments with coral colonies (Fong, Smith & Wartian, 2006). Although some CCA species may be deleterious to corals, these organisms generally facilitate coral growth in heterogenous landscapes (Price, 2010). In our study, the strong effect of cyanobacteria may have obliterated the statistical effect of CCA on coral growth trends. Similarly, temperature was not a significant predictor of coral growth, even though coral growth declined after the 2010 temperature spike (Figs. 2 and 5).

In Abrolhos, roving herbivorous fish are more abundant in offshore protected reefs (Francini-Filho & Moura, 2008). From 2003 to 2008, Francini-Filho et al. (2013) monitored the region’s benthic assemblages and failed to detect significant temporal changes in foliose macroalgae cover in both inshore and offshore reefs, despite recording consistent cross-shelf differences in macroalgal abundance. Although it is unclear whether the higher inshore macroalgal abundance (10–20% of reef cover) is a stable phase related to a long-standing high turbidity background, or a contemporary response to overfishing (Freitas et al., 2011), increased macroalgal abundance implies a higher contact frequency with corals (Grillo, Bonaldo & Segal, 2018). Poor land use and coastal development enhances sedimentation and reduce the overall vitality of reef systems, which is generally inversely related to distance offshore (Dutra, Kikuchi & Leão, 2006). However, our results challenge the idea that high macroalgal cover is always associated with compromised coral health, as baselines for turbid zone reefs may differ sharply from those of coral-dominated reefs that dwell under more oligotrophic conditions (Rogers, 1990; Kleypas, 1996). Indeed, our results add to the growing evidence of increased physiological plasticity of South Atlantic corals in terms of fecundity (Pires, Segal & Caparelli, 2011) and photoacclimation (Sugget et al., 2012) under marginal conditions. In Abrolhos, instead of being additive or synergistic (e.g., Diaz-Pulido et al., 2011), the effects of turbidity and cyanobacteria were antagonistic.

Conservation concerns are higher in inshore unprotected reefs, due to their proximity to anthropogenic stressors and overfishing (e.g., Francini-Filho & Moura, 2008). However, coral decline was faster in offshore reefs, which may be more vulnerable to stressors that operate at larger spatial scales, such as light and temperature anomalies. The anomalous growth of cyanobacteria, leading to toxic amounts of cyanotoxins (Rastogi, Madamwar & Incharoensakdi, 2015), are often attributed to temperature anomalies and anthropogenic impacts such as nutrient input. However, as we have demonstrated, distance offshore and no-take zoning are not always sufficient to keep reefs from harmful cyanobacterial blooms. For instance, despite the significantly higher fish abundances inside the offshore no-take zone (Francini-Filho & Moura, 2008), roving herbivorous were never recorded eating cyanobacteria mats and tufts. Avoiding coral decline goes well beyond no-take zoning and should include water quality control and climate change mitigation, as well as appropriate local baselines for long term monitoring, as health indicators are geographically variable and context dependent.

## Conclusion

Our results add to the growing evidence that turbid zone reefs present unique functional properties that challenge the current models explaining the global decline of coral reefs, which are largely based on DOC, disease, algae, and microorganisms. Here, we show that the growth of one of the main reef builders of the Abrolhos reefs, *M. braziliensis*, was positively influenced by turbidity, which is greater nearshore. On the other hand, filamentous cyanobacteria were the most aggressive coral competitors. The negative effect of filamentous cyanobacteria was detected both in unprotected nearshore reefs and in no-take offshore reefs with less turbidity. Besides this significant negative effect of filamentous cyanobacteria detected from one decade of field records, we presented evidence of an inhibitory effect of their exudates over *ex hospite* zooxanthellae (*Symbiodinium*) isolated from local corals. We emphasize the importance of building local baselines for long-term monitoring, as health indicators are geographically variable and context dependent. Avoiding coral decline should go well beyond no-take zoning and includes a longer road that passes through water quality control and climate change mitigation.

##  Supplemental Information

10.7717/peerj.5419/supp-1 Table S1 Results of pairwise tests contrasting between-year changes in the area of live coral tissue

10.7717/peerj.5419/supp-2 Table S2 Raw data (coral area and contact length with surrounding organisms)

10.7717/peerj.5419/supp-3 Table S3 Raw data (environmental variables)

## Additional Information and Declarations

### Field Study Permissions

The following information was supplied relating to field study approvals (i.e., approving body and any reference numbers): Permits for field study were granted by the Abrolhos National Park (SISBIO permits number 49667-1, 50872-2, 51670-2).

### Data Availability

The following information was supplied regarding data availability: The raw data are provided in the Supplemental Files.
