# Supplementary material for: Long-term effects of competition and environmental drivers on the growth of the endangered coral Mussismilia braziliensis (Verril, 1867)
Source: PeerJ. 2018 Aug 10;6:e5419. doi: 10.7717/peerj.5419 (PMC6089213; doi:10.7717/peerj.5419)
Supplement: Table S1 [file peerj-06-5419-s001.docx]

Table S1. Results of pairwise tests contrasting between-year changes in the area of live coral tissue.

| PAB (offshore site) |  |  |
| --- | --- | --- |
| Year | t | p |
| 2008, 2009 | 1.1555 | 0.2713 |
| 2008, 2012 | 0.9671 | 0.3498 |
| 2008, 2013 | 1.4535 | 0.1707 |
| 2008, 2014 | 0.858 | 0.3967 |
| 2008, 2015 | 1.458 | 0.1621 |
| 2008, 2016 | 2.0453 | 0.0623 |
| 2009, 2012 | 0.0004 | 0.9977 |
| 2009, 2013 | 0.9286 | 0.4272 |
| 2009, 2014 | 1.9859 | 0.071 |
| 2009, 2015 | 0.6805 | 0.5228 |
| 2009, 2016 | 1.5429 | 0.1559 |
| 2012, 2013 | 0.8645 | 0.4312 |
| 2012, 2014 | 1.7547 | 0.1042 |
| 2012, 2015 | 0.528 | 0.6062 |
| 2012, 2016 | 1.2212 | 0.2312 |
| 2013, 2014 | 2.0087 | 0.0564 |
| 2013, 2015 | 0.5646 | 0.6041 |
| 2013, 2016 | 0.126 | 0.9058 |
| 2014, 2015 | 2.1859 | 0.0448 |
| 2014, 2016 | 2.6795 | 0.0121 |
|  |  |  |
| PLES (coastal site) |  |  |
| Year | t | P |
| 2009, 2012 | 3.0073 | 0.017 |
| 2009, 2013 | 1.5085 | 0.176 |
| 2009, 2014 | 1.8853 | 0.098 |
| 2009, 2015 | 2.1628 | 0.042 |
| 2009, 2016 | 2.6358 | 0.023 |
| 2012, 2013 | 2.2437 | 0.04 |
| 2012, 2014 | 1.4436 | 0.173 |
| 2012, 2015 | 1.5861 | 0.139 |
| 2012, 2016 | 0.3372 | 0.726 |
| 2013, 2014 | 0.6868 | 0.514 |
| 2013, 2015 | 0.8225 | 0.458 |
| 2013, 2016 | 2.071 | 0.062 |
| 2014, 2015 | 0.0318 | 0.974 |
| 2014, 2016 | 1.5336 | 0.155 |
| 2015, 2016 | 1.589 | 0.137 |
| 2009, 2012 | 3.0073 | 0.017 |
